# Research note: Pool sequencing nominates *MITF* as a key candidate gene for white-belted feathers in chickens

**DOI:** 10.1016/j.psj.2025.106033

**Published:** 2025-10-30

**Authors:** Xunhe Huang, Yiqi Jiang, Ningying Zheng, Huatao Liu, Zhuoxian Weng, Jintian Wen, Jingyi Li, Cheng Ma

**Affiliations:** a Guangdong Provincial Key Laboratory of Conservation and Precision Utilization of Characteristic Agricultural Resources in Mountainous Areas, Guangdong Innovation Centre for Science and Technology of Wuhua Yellow Chicken, School of Life Sciences, Jiaying University, 514015 Meizhou, China; b Key Laboratory of Agricultural Animal Genetics, Breeding and Reproduction of Ministry of Education, College of Animal Science and Technology and College of Veterinary Medicine, Huazhong Agricultural University, 430070 Wuhan, China; c Department of Medical Biochemistry and Microbiology, Uppsala University, Uppsala, Sweden

**Keywords:** White-belted phenotype, Pool-seq, *MITF*, Melanogenesis, Chicken

## Abstract

A rare variant of the Wuhua yellow chicken rooster exhibits a distinct plumage phenotype characterized by a white belt across the dorsal region and wing shoulder. This variant provides a valuable model for studying regional pigmentation genetics. Using pool sequencing, we identified candidate genes via high-depth sequencing (average 52 × coverage) of DNA pools from 35 brown-belted and 35 white-belted roosters. Integrated analysis of allele frequency differences, Fisher’s exact test, and population differentiation revealed 49 shared candidate genes, with 23 concentrated in a 3.83 Mb region on chromosome 12 (13.12–16.95 Mb). Notably, *MITF* and two adjacent genes exhibited strong selection signals across all methods. Functional enrichment analyses highlighted significant terms related to melanocyte differentiation and the melanogenesis pathway, with *MITF* serving as a core regulator. Comparative genomic evidence further supported the conserved role of *MITF* in pigment deposition across vertebrates. We propose *MITF* as the primary genetic regulator of the white-belted phenotype, potentially modified by other genes such as *PRICKLE2*. This study provides insights into the genetic architecture of regional depigmentation and underscores the role of *MITF* in avian plumage patterning.

## Introduction

Plumage color diversity is an economically significant trait in poultry that is primarily determined by pigment-based mechanisms and structural characteristics. Feather coloration in chickens is mainly influenced by melanins, which are endogenously synthesized pigments derived from tyrosine metabolism and are categorized into dark eumelanin and reddish-brown pheomelanin (Solano, 2016). The diversity of chicken plumage patterns depends on the relative proportion and spatial distribution of these melanins within feathers (Roulin and Ducrest, 2013).

Melanin synthesis is primarily regulated by the microphthalmia-associated transcription factor (***MITF***) gene, which serves as a master regulator of melanogenesis. *MITF* controls the development, survival, and differentiation of melanocytes by activating key melanogenic enzymes, such as tyrosinase (***TYR***) and tyrosinase-related protein 1 (***TYRP1***), thereby directly governing the production of eumelanin and pheomelanin (Andersson et al., 2020). Mutations or structural variations in *MITF* often lead to impaired melanocyte function and localized or systemic pigment loss. For instance, in ducks, aberrant *MITF* expression correlates with reduced melanin deposition and white plumage phenotypes (Wang et al., 2024). Similarly, *MITF* defects are associated with depigmentation in diverse vertebrate species, highlighting its essential and evolutionary conserved role in pigment biology (Mcnamara et al., 2021).

Wuhua yellow chicken, a local breed native to Guangdong, China, is a typical yellow-feathered chicken known for its uniform yellow body plumage and white tail feathers. However, a notable variant exists in roosters, where a distinct depigmented (white) belt appears across the back and wing shoulder. Although the genetic basis of overall plumage color has been extensively studied, mechanisms governing regional pigment loss, especially the belted pattern in chickens, remain poorly understood. Thus, this phenotype provides a compelling model for identifying genetic determinants of regional pigment loss.

To unravel the genetic basis of this trait, we employed pool sequencing (**pool-seq**), population genomic analyses, and functional enrichment. Our integrated approach nominated *MITF* as the top candidate. Given its central role in melanogenesis and established association with similar phenotypes across species, we propose *MITF* as a strong candidate gene for the white-belted phenotype in chickens.

## Materials and methods

### Ethics statement and sampling

**Fig. 1:**
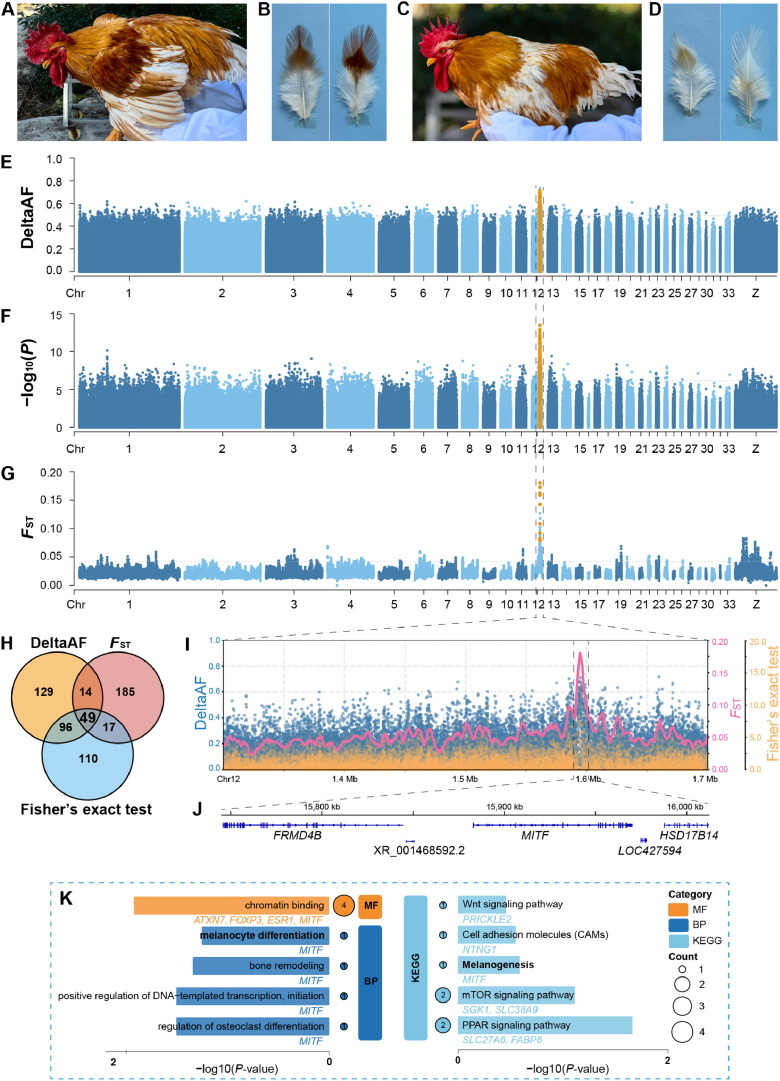
Genetic analysis of the white-belted phenotype in Wuhua yellow chicken. (A) Phenotype of a brown-belted rooster. (B) Feather details of the back (left) and wing shoulder (right) from a brown-belted rooster. (C) Phenotype of a white-belted rooster. (D) Feather details of the back (left) and wing shoulder (right) from a white-belted rooster. (E) Manhattan plot of the DeltaAF values across chromosomes, with the *MITF* gene highlighted in yellow. (F) Manhattan plot of Fisher’s exact test [−log₁₀(*P*)] results across chromosomes, with the *MITF* gene highlighted in yellow. (G) Manhattan plot of *F*_ST_ values across chromosomes, with the *MITF* gene highlighted in yellow. (H) Venn diagram illustrating the overlap of signals detected by the DeltaAF, Fisher’s exact test, and *F*_ST_ analyses. (I) Local Manhattan plot of selection signals on chromosome 12 (13.12–16.95 Mb), with scatter points representing DeltaAF (dark blue), Fisher’s exact test (orange), and *F*_ST_ (coral pink). The top candidate region is indicated. (J) Gene structure of *MITF* within the top candidate region, with the most significant selection signal indicated. (K) Functional enrichment analysis. Significantly enriched Gene Ontology (GO) terms and Kyoto Encyclopedia of Genes and Genomes (KEGG) pathways are shown, with bar length indicating statistical significance (-log₁₀(*P*-value)) and point size representing gene count. Colors denote the GO functional categories molecular function (MF), and biological process (BP) and KEGG pathways. Key melanin-related terms involving *MITF* are highlighted.

All animal procedures followed guidelines approved by the Institutional Animal Care and Use Committee of Jiaying University (protocol number: JYDWLL2024-12). Sampling was conducted at Fengdu Integrated Poultry Farm in Meizhou, China. A total of 70 thirty-week-old Wuhua yellow chicken roosters exhibiting the rare belted phenotype were carefully selected from a conservation population. This cohort included 35 brown-belted individuals, which exhibited brown back and wing feathers with no abnormal white feather distribution on the saddle region (Fig. 1A and B), and 35 white-belted individuals, which were characterized by white back and wing feathers alongside normal yellow coloration on the saddle region (Fig. 1C and D).

### DNA preparation and quality control

Approximately 1 mL of venous blood was collected from the wing vein of each bird using EDTA-K₂ tubes and stored at −40°C. Genomic DNA was extracted using the HiPure Universal DNA Kit (Magen Biotechnology, China). DNA quality was assessed by 1% agarose gel electrophoresis and quantified with a NanoDrop 2000 spectrophotometer (Thermo Fisher Scientific, USA). Qualified DNA was normalized to 100 ng/μL for library preparation.

### Pool-seq library construction and sequencing

Equal amounts of DNA from each group were pooled to construct two pools representing each phenotype. Libraries were prepared with the MGIEasy PCR-Free DNA Kit (MGI Tech Co., Ltd., Shenzhen, China) following the manufacturer's instructions, with an average insert size of 350 bp. Paired-end sequencing (150 bp reads) was performed on the DNBSEQ-T7 platform at approximately 50 × genome coverage.

### Bioinformatic processing and alignment

Raw sequencing data underwent rigorous quality control with FastQC v0.12.1. Clean reads were aligned to the chicken reference genome GRCg6a (GenBank assembly accession: GCA_000002315.5) using BWA-MEM v0.7.18. Duplicate marking and file formatting were performed using Samtools v1.21 and Picard tools v3.4.0. The mpileup command generated a merged mpileup file, converted to a synchronized format using mpileup2sync.jar from PoPoolation2 (Kofler et al., 2011) for downstream analyses.

### Population genomic analyses

We used PoPoolation2 for three analyses: allele frequency differences (**DeltaAF**), Fisher's exact test and fixation index (***F*_ST_**) estimation (Kofler et al., 2011). Uniform filtering parameters included: minimum allele count of 4, minimum coverage of 30 reads per pool, and maximum coverage capped at the top 2% of sites. *F*_ST_ analysis used a sliding window of 50 kb with a 10 kb step. Significance was defined using percentile ranks: top 0.01% for DeltaAF and Fisher's exact test (−log₁₀(*P*)), and top 1% windows for *F*_ST_.

### Functional annotation and enrichment analysis

Manhattan plots were generated with CMplot. Significant SNPs were annotated using Bedtools v2.27.0 to retrieve Ensembl IDs and gene symbols. Functional enrichment analysis was performed using KOBAS for both Gene Ontology (**GO**) terms and Kyoto Encyclopedia of Genes and Genomes **(KEGG**) pathways. Terms related to pigment synthesis and regulation were identified, and candidate genes were selected based on functional relevance.

## Results and discussion

### High-quality sequencing data and genetic analysis

The pool-seq approach generated robust datasets, with average sequencing depths of 52.26 × and 52.39 × for brown- and white-belted pools, respectively, and a mean coverage of 97.31%. After quality control, 12,386,451 single nucleotide polymorphisms (**SNPs**) were obtained. Using three complementary approaches, we identified significant selection signals: DeltaAF (top 0.01%; cutoff > 0.491) revealed 1,215 SNPs (288 Ensembl genes), Fisher's exact test (top 0.01%; cutoff > 6.144) detected 1,229 SNPs (272 genes), and *F*_ST_ (top 1% windows; cutoff > 0.042) uncovered 998 candidate regions (265 genes) (Fig. 1E–G). Intersection analysis of the three methods yielded 49 shared Ensembl genes distributed across multiple chromosomes, with 23 genes concentrated within a 3.83 Mb region on chromosome 12 (13.12–16.95 Mb) (Fig. 1H; Table 1). Among these, *MITF* and two neighboring genes *FRMD4B* and *HSD17B14* exhibited the strongest signals across all methods, with *MITF* showing the highest proportion of significant SNPs relative to its total SNP content (113/1005, ∼11.24%), thus identifying *MITF* as the top-ranked candidate (Fig. 1I, J; Table 1).

**Table 1:** Candidate genes under selection identified by integrated analysis of pool sequencing data.

Chr	Location	Ensembl gene	Symbol gene	DeltaAF ^a^	Fisher’s exact test ^b^	*F*_ST_
2	38854063–39545916	ENSGALG00000011439	*-*	0.505 (1/7756)	7.804 (1/7756)	0.042
3	49053965–49241576	ENSGALG00000012973	*ESR1*	0.504 (1/1164)	6.773 (1/1164)	0.045–0.049
3	55846631–55911966	ENSGALG00000013962	*HBS1L*	0.518–0.525 (2/517)	6.749 (1/517)	0.051–0.063
3	56205535–56302921	ENSGALG00000013971	*SGK1*	0.538 (1/1324)	6.260 (1/1324)	0.044
4	6565250–6989919	ENSGALG00000006851	*PCDH11X*	0.551 (1/3311)	6.997 (1/3311)	0.042–0.049
6	11386546–11421551	ENSGALG00000031604	*-*	0.509–0.540 (2/501)	6.811–7.349 (2/501)	0.043–0.047
6	9774397–9819482	ENSGALG00000003165	*CCDC6*	0.525 (1/684)	7.168 (1/684)	0.043–0.047
8	1215190–1359430	ENSGALG00000031122	*NTNG1*	0.503 (1/1146)	6.962–8.748 (4/1146)	0.048–0.049
8	20538514–20684008	ENSGALG00000010083	*ST3GAL3*	0.496–0.576 (3/2263)	6.385–6.963 (3/2263)	0.044
8	20919007–20982770	ENSGALG00000045795	*-*	0.506–0.541 (2/907)	6.501 (1/907)	0.043
8	30055243–30137386	ENSGALG00000011379	*SLC44A5*	0.538 (1/302)	6.384 (1/302)	0.044–0.045
11	12824211–12973016	ENSGALG00000005319	*CDH8*	0.533 (1/1186)	6.242 (1/1187)	0.045–0.064
12	13123560–13498453	ENSGALG00000007177	*PTPRG*	0.495–0.572 (9/4471)	6.158–7.768 (6/4471)	0.042–0.048
12	13553845–13726591	ENSGALG00000007268	*CADPS*	0.515–0.543 (9/2462)	6.147–8.053 (13/2462)	0.043–0.047
12	13943984–14003991	ENSGALG00000007302	*ATXN7*	0.524 (1/865)	6.792–7.098 (2/865)	0.043
12	14033841–14122269	ENSGALG00000007332	*PRICKLE2*	0.539–0.666 (5/1476)	6.254–11.008 (4/1476)	0.043–0.052
12	14132885–14195481	ENSGALG00000041750	*ADAMTS9*	0.505–0.589 (7/1016)	6.235–9.010 (7/1016)	0.047–0.058
12	14398620–14690794	ENSGALG00000007431	*MAGI1*	0.495–0.654 (3/4674)	6.177–8.755 (31/4674)	0.044–0.065
12	14847918–14926384	ENSGALG00000007483	*LRIG1*	0.514–0.547 (5/1088)	6.665–8.092 (4/1088)	0.044–0.055
12	15014839–15023143	ENSGALG00000007569	*KBTBD8*	0.529–0.539 (2/113)	6.286–6.660 (2/113)	0.048–0.073
12	15030285–15057873	ENSGALG00000007596	*MCT2L*	0.527–0.596 (6/523)	6.233–8.178 (6/523)	0.049–0.073
12	15139287–15242142	ENSGALG00000007652	*SUCLG2*	0.532–0.663 (16/1738)	6.240–9.972 (20/1738)	0.043–0.068
12	15332071–15534822	ENSGALG00000049667	*TAFA1*	0.494–0.611 (19/2947)	6.260–9.710 (21/2947)	0.042–0.073
12	15594740–15637018	ENSGALG00000042228	*-*	0.508–0.587 (4/595)	6.205–6.624 (4/595)	0.051–0.060
12	15663150–15690673	ENSGALG00000013406	*EOGT*	0.497–0.603 (8/415)	6.483–10.372 (5/415)	0.051–0.068
12	15693136–15706614	ENSGALG00000013408	*TMF1*	0.543 (1/180)	7.908 (1/180)	0.053–0.069
12	15707287–15721052	ENSGALG00000013410	*UBA3*	0.493 (1/185)	6.169 (1/185)	0.061–0.069
12	15721935–15731462	ENSGALG00000038904	*ARL6IP5*	0.523–0.616 (6/142)	8.121–9.955 (7/142)	0.061–0.069
12	15745789–15810437	ENSGALG00000037791	*FRMD4B*	0.505–0.670 (7/1213)	6.184–11.193 (11/1213)	0.049–0.071
12	15882966–15968266	ENSGALG00000039583	*MITF*	0.492–0.712 (112/1005)	6.193–13.460 (113/1005)	0.075–0.180
12	15983524–16011478	ENSGALG00000007727	*HSD17B14*	0.499–0.658 (13/294)	6.171–11.870 (16/294)	0.092–0.180
12	16053940–16058208	ENSGALG00000007741	*-*	0.525–0.539 (3/60)	6.257–6.818 (3/60)	0.059–0.092
12	16307942–16503337	ENSGALG00000007769	*FOXP3*	0.509–0.583 (8/1912)	6.426–6.912 (4/1912)	0.044–0.080
12	16898430–16917447	ENSGALG00000007804	*GXYLT2*	0.497–0.525 (2/393)	6.399–7.014 (2/393)	0.042–0.043
12	16918018–16950486	ENSGALG00000007810	*PPP4R2*	0.494–0.520 (3/520)	6.445–8.357 (3/520)	0.043–0.044
13	8593917–8632025	ENSGALG00000001445	*FABP6*	0.542–0.573 (4/726)	7.134–9.397 (4/726)	0.046–0.054
19	801748–844553	ENSGALG00000001042	*MTMR4*	0.537 (1/626)	6.555 (1/626)	0.043–0.045
19	9375787–9405036	ENSGALG00000041802	*LYRM9*	0.493 (1/98)	6.347 (1/98)	0.048–0.069
23	355962–372948	ENSGALG00000043185	*MATN1*	0.502 (1/200)	6.983 (1/200)	0.043
24	5145236–5215773	ENSGALG00000007144	*SIK3*	0.503–0.504 (2/903)	6.590–7.076 (2/903)	0.044
Z	16643589–16666277	ENSGALG00000013548	*GZMA*	0.524 (1/167)	7.090 (1/167)	0.046–0.078
Z	16648999–16663409	ENSGALG00000013546	*GZMK*	0.524 (1/120)	7.090 (1/120)	0.046–0.077
Z	16883916–16926017	ENSGALG00000014712	*SLC38A9*	0.530 (1/209)	6.833 (1/209)	0.048–0.083
Z	16981247–17014499	ENSGALG00000030800	*-*	0.500–0.515 (2/200)	6.158 (1/200)	0.043–0.078
Z	17026875–17060827	ENSGALG00000014716	*IL6ST*	0.504–0.556 (2/209)	6.480 (1/209)	0.048–0.078
Z	22593889–22715686	ENSGALG00000004437	*LHFPL2*	0.514–0.517 (2/927)	6.353 (1/927)	0.046–0.067
Z	22721122–22759474	ENSGALG00000004425	*SCAMP1*	0.519–0.567 (2/244)	6.677 (1/244)	0.048–0.060
Z	35092121–35350353	ENSGALG00000015126	*TRPM3*	0.500–0.560 (5/2115)	6.389 (7/2115)	0.042–0.054
Z	45721101–45760913	ENSGALG00000000184	*SLC27A6*	0.493 (1/279)	6.559–6.727 (2/279)	0.057–0.067

Note: ^a^ DeltaAF values are shown with the ratio of significant to the total SNPs within the gene (significant/total) indicated in parentheses. ^b^ Values for Fisher's exact test are presented as −log₁₀(*P*), with the corresponding ratio of significant to the total SNPs within the gene (significant/total) in parentheses.

### Functional enrichment supports MITF's central role

GO analysis revealed significant enrichment in terms directly related to pigmentation, notably melanocyte differentiation (GO:0030318, *P* = 0.045), with *MITF* as a key regulator (Fig. 1K). KEGG analysis highlighted melanogenesis (KEGG:gga04916, *P* = 0.224), where *MITF* is a core transcriptional regulator (Fig. 1K). Enrichment of the Wnt signaling pathway (KEGG:gga04310, *P* = 0.316) suggested a potential role for *PRICKLE2*. Collectively, these findings provide strong evidence that *MITF* is the foremost candidate gene for this phenotype.

### Comparative genomic evidence across vertebrate species

The critical role of *MITF* in pigmentation is profoundly conserved across vertebrates. This variation drives a wide spectrum of phenotypes, from complete depigmentation to intricate spatial patterns. In ducks, *MITF* variations impair melanin synthesis and cause white plumage (Zhou et al., 2018). Similarly, studies in Putian black ducks further demonstrated reduced *MITF*-*M* promoter activity and melanin deficiency (Lin et al., 2024). Beyond birds, *MITF* loss-of-function mutations cause white spotting in swamp buffalo (Dai et al., 2024) and cattle (Hofstetter et al., 2019). This repeated implication across species strongly supports *MITF* as the genetic determinant of the white-belted phenotype in Wuhua yellow chickens.

### MITF as the principal determinant with potential modifying factors

*MITF* functions as a master transcriptional regulator of melanin synthesis by controlling downstream targets, including *TYR, TYRP1*, and *DCT*; moreover, *MITF* is regulated by *MC1R, ASIP*, and *SOX10* (Levy et al., 2006). It mediates multiple aspects of melanocyte biology, including survival, migration, proliferation, and melanogenesis. Despite the implication of other genes (e.g., *PRICKLE2*), their pigmentation roles are likely indirect. Thus, although potential modifying effects from other genes remain possible, our findings firmly establish *MITF* as the principal genetic determinant. To validate this robust genetic association, the functional role of *MITF* should be confirmed through transcriptomic comparisons and subsequent experimental validation.

In summary, our findings elucidate how the conserved *MITF*-regulated melanogenesis network can be modulated to produce a distinct spatial patterning in chickens. This insight contributes to a broader understanding of vertebrate pigment patterning, and carries important implications for evolutionary developmental biology and suggests potential routes for selective breeding in poultry.

## Funding

This work was supported by the Peak Talent Program of Jiaying University (2022RC45), the Key Discipline Construction Project of Guangdong Provincial Department of Education (2022ZDJS088), and Natural Science Research Project of Jiaying University (322E0102).

## CRediT authorship contribution statement

**Xunhe Huang:** Conceptualization, Data curation, Formal analysis, Funding acquisition, Investigation, Methodology, Project administration, Visualization, Writing – original draft, Writing – review & editing. **Yiqi Jiang:** Data curation, Formal analysis, Resources, Writing – review & editing. **Ningying Zheng:** Data curation, Resources, Writing – review & editing. **Huatao Liu:** Data curation, Resources, Writing – review & editing. **Zhuoxian Weng:** Data curation, Funding acquisition, Resources, Writing – review & editing. **Jintian Wen:** Visualization, Writing – review & editing. **Jingyi Li:** Methodology, Writing – review & editing. **Cheng Ma:** Writing – review & editing.

## Disclosures

The authors declare that they have no known competing financial interests or personal relationships that could have appeared to influence the work reported in this paper.
